# Updating the Comparative Evidence on Second‐Generation Antipsychotic Use With Schizophrenia

**DOI:** 10.1176/appi.prcp.20200004

**Published:** 2020-10-16

**Authors:** Marian S. McDonagh, Tracy Dana, Shelley Selph, Emily B. Devine, Amy Cantor, Christina Bougatsos, Ian Blazina, Sara Grusing, Rongwei Fu, Daniel W. Haupt

**Affiliations:** ^1^ Pacific Northwest Evidence‐Based Practice Center Portland Oregon; ^2^ Department of Medical Informatics and Clinical Epidemiology; ^3^ Department of Family Medicine; ^4^ School of Public Health; ^5^ Department of Psychiatry; ^6^ Oregon Health and Science University Portland; ^7^ Department of Pharmacy and Comparative Health Outcomes Police and Economics Institute University of Washington Seattle

## Abstract

**Objective:**

The objective of this study was to conduct a systematic review of literature comparing second‐generation antipsychotics (SGAs) with each other and with first‐generation antipsychotics (FGAs) in treating schizophrenia.

**Methods:**

MEDLINE, the Cochrane Library, and PsycINFO databases were searched through January 2020. Following standard methods, recent high‐quality systematic reviews of each drug comparison and subsequently published primary studies were included to update the meta‐analyses with any new data. Two reviewers independently conducted study selection, abstraction, and quality assessment.

**Results:**

Two systematic reviews and 29 newer trials (total of 162 trials of SGAs, N=53,861; 116 trials of SGAs versus FGAs, N=119,558) were included. Most trials were of fair quality, industry‐funded, and included older SGAs and a few recently approved SGAs (asenapine, lurasidone, iloperidone, cariprazine, brexpiprazole and long‐acting injection [LAI] formulations of aripiprazole and paliperidone). Older SGAs had similar effects on function, quality of life, mortality, and adverse event incidence, although clozapine improved symptoms more than most other drugs and olanzapine and risperidone were superior to some other drugs. Olanzapine, risperidone, ziprasidone, and aripiprazole performed similarly on outcomes of benefit compared with haloperidol. Risperidone LAI and olanzapine resulted in fewer withdrawals due to adverse events, but risk of diabetes increased with olanzapine. Haloperidol had greater incidence of adverse events than did olanzapine and risperidone, but similar effects on other outcomes.

**Conclusions:**

Most comparative evidence favored older SGAs, with clozapine, olanzapine, and risperidone superior on more outcomes than other SGAs. Older SGAs had similar benefits as haloperidol but with fewer adverse events.

## HIGHLIGHTS

- Use of the second‐generation antipsychotics olanzapine, aripiprazole, risperidone, quetiapine, and ziprasidone resulted in similar function, quality of life, mortality, and overall incidence of adverse events.
- Core illness symptoms improved more with olanzapine and risperidone than asenapine, quetiapine, or ziprasidone, and more with paliperidone than lurasidone or iloperidone.
- Haloperidol showed similar benefits but more adverse events than did olanzapine or risperidone.

Schizophrenia is a chronic mental health condition that most often manifests in early adulthood and can lead to episodic and varying levels of disability. The most recent version of the *DSM‐5* (1) reflects the *DSM‐III* and *DSM‐IV* diagnostic criteria for schizophrenia, simplified and clarified, with no change in the defined patient population among the editions (2). *DSM‐5* criteria define schizophrenia as the presence of two or more of five core symptoms (delusions, hallucinations, disorganized speech, grossly disorganized or catatonic behavior, and negative symptoms). At least one of the symptoms must be delusions, hallucinations, or disorganized speech, and symptoms must be present for at least 6 months. Lifetime prevalence is reported to be 0.3% to 0.7%, with onset most commonly occurring in late adolescence through the third decade (3). Differential diagnosis is broad and includes delineation from mood disorders (bipolar disorder or major depressive disorder) with psychotic features and substance and/or medication‐induced psychotic disorders. The course of schizophrenia varies; approximately 20% of patients may experience significant improvement, including, in some cases, full recovery (4). However, most patients experience some degree of social and occupational difficulty as well as need for support in daily living. Recent research and practice have focused on early intervention with first‐episode psychosis, which has demonstrated promise toward improving outcomes, reducing longer‐term disability, and improving the likelihood of full recovery (5).

A mainstay of early intervention is pharmacologic therapy (6). Antipsychotic medications act primarily via dopaminergic antagonism and can result in meaningful improvements in symptoms. Ideally, improvements in symptoms translate to long‐term, clinically relevant changes in other outcome areas, with limited, manageable adverse effects. Although efficacy trials of antipsychotics conducted for regulatory approval are limited to measurement of changes in symptoms, measurement of recovery‐oriented outcomes (e.g., remission) reflecting improvement in social and occupational functioning, and ultimately quality of life, are necessary. Historically, there has been uncertainty regarding the impact of antipsychotic drugs on long‐term patient‐centered outcomes, such as consistent employment, successful interpersonal relationships, maintenance of independent living, and the absolute and relative risk of serious long‐term adverse effects (e.g., tardive dyskinesia and diabetes). Many patients discontinue their prescribed antipsychotic medication, and discontinuation rates and time to discontinuation vary by treatment and patient characteristics. Older, first‐generation antipsychotics (FGAs), such as haloperidol, have proven efficacy in reducing symptoms, but adverse effects, such as extrapyramidal symptoms and sometimes tardive dyskinesia, often limit long‐term use. Second‐generation antipsychotics (SGAs) were introduced as having efficacy equal to or better than that of FGAs, particularly for negative symptoms, and possibly lower risk for the adverse events that limited use of FGAs (7, 8, 9). SGAs, however, also have potentially serious long‐term adverse effects (e.g., cardiovascular and endocrinological effects) that make their overall risk‐benefit profile less clear‐cut. Additionally, the specific mechanisms of action (e.g., interaction with dopamine receptors) and adverse effect profiles vary across the SGAs. Twelve SGAs (with 22 formulations) are currently marketed in the United States, along with several FGAs. Given the availability of newer drugs and formulations, and the need to select specific treatments for individual patients with schizophrenia, updated evaluation of the comparative evidence for a range of outcomes, including symptoms, function, and quality of life, associated with these drugs is important.

In this review, we aim to update and summarize the key findings of a systematic review on the comparative effectiveness of SGAs versus each other and versus FGAs as treatments for patients with schizophrenia. These findings are part of a broader report funded and published by the Agency for Healthcare, Research and Quality (AHRQ) (10). The review topic was nominated by the American Psychiatric Association (APA) to provide evidence in support of updating guidelines on treatments for schizophrenia. The full report includes evidence on psychosocial treatments and is much more detailed in both methods and results reporting but is less accessible for most readers.

## METHODS

We adhered to AHRQ guidance for methodology in comparative effectiveness reviews (11, 12). The scope of this review was based on consultation with an APA guideline development group and was refined through consultation with experts. The Oregon Health and Science University Institutional Review Board determined that systematic reviews conducted by the Evidence‐Based Practice Center are not human subjects research. The protocol has been published on the AHRQ Effective Health Care website. The work was conducted between January 2016 and March 2017, with updating in January 2020. The draft of that report was revised based on comments from invited reviewers and comments received through public posting prior to finalization. In this review, we have updated the findings with new evidence published since the AHRQ report.

### Literature Search and Study Selection

A research librarian searched MEDLINE, the Cochrane Central Register of Controlled Trials, the Cochrane Database of Systematic Reviews, PsycINFO, and web pages of organizations that fund systematic reviews through February 2017 for the AHRQ report, and conducted updated searches in MEDLINE through January 2020 for this manuscript. In accordance with AHRQ guidance to improve efficiency (13), we first searched for recent, high‐quality systematic reviews that had a similar scope to our AHRQ review; that is, reviews of randomized controlled trials (RCTs) of at least 12 weeks duration that directly compared SGAs with each other or an FGA for patients with schizophrenia. We included one review for any given drug comparison‐outcome pair, so that data from any given study were included only once. We then searched for and included RCTs that were published after we had searched for the included systematic reviews. We requested information from pharmaceutical manufacturers and searched reference lists of the included studies. Key outcomes were clinical and patient centered (i.e., focused on health outcomes) and were selected based on input from an expert panel. These outcomes were functional quality of life improvements, reductions in self‐harm, treatment discontinuation, symptom‐related outcomes, withdrawals due to adverse events, and significant adverse events (including deaths). Search results were independently screened for eligibility by two reviewers, with disagreements resolved by achieving consensus. Complete search strategies and lists of included studies and studies excluded after full‐text review can be found in the appendices of the full report (10).

### Study Quality and Evidence Synthesis

By using predefined criteria (10), we assessed the quality of RCTs and systematic reviews. We evaluated the RCTs using methods developed by the Drug Effectiveness Review Project (14). We assessed systematic reviews by using A Measurement Tool to Assess Systematic Reviews (AMSTAR), a quality‐rating instrument (15). We rated studies as good, fair, or poor. Studies deemed poor quality, with multiple flaws, were considered less reliable and were not synthesized with higher‐quality studies. Study quality was independently assessed by two reviewers, with disagreements resolved by achieving consensus.

Meta‐analyses were considered, depending on the data available and the similarity among studies in design, patient populations, interventions, and outcomes. Meta‐analyses including systematic reviews were updated with data from newer trials where possible, and if no new data were available, the results of the meta‐analyses in the included reviews were reported. We used the DerSimonian and Laird random‐effects model for pairwise meta‐analyses. We updated the pair‐wise meta‐analyses by using StatsDirect, version 3.0 (Camcode). Statistical heterogeneity was assessed using the I^2^ statistic (16). We updated network meta‐analyses of symptom response, overall discontinuation of drug, and withdrawal due to adverse events that were initially conducted in the included systematic review of SGAs (17) by using Stata/SE, version 14.1 (StataCorp), and the Bayesian model was performed with OpenBUGS, version 3.2.3 (18, 19). We controlled the analysis for variation in study duration, mean daily dose levels (low, medium, and high), and whether studies enrolled patients with a first episode of schizophrenia or whose symptoms were resistant to prior treatment. We defined response as 20% improvement on the Positive and Negative Syndrome Scale (PANSS) or by using other scales, such as the Clinical Global Impression‐Improvement scale (CGI‐I) and combinations of these scales). Where results were not combinable in a meta‐analysis because of heterogeneous populations, interventions, comparators, or outcome measures, or where an important outcome was reported for a given comparison in only a single trial, we report individual trial results. There were not enough new data to update meta‐analyses of SGAs versus FGAs.

Strength of the body of evidence for each key outcome was assessed by using the approach outlined for AHRQ evidence‐based practice centers by evaluating the following domains: study limitations (i.e., cumulative study quality), consistency, directness of evidence, and precision of estimates (12, 20). We assigned overall grades of high, moderate, low, or insufficient based on the domain evaluations: high strength of the body of evidence indicated confidence in the estimate of effect, whereas moderate and low reflected lower levels of confidence such that future evidence could alter the results. A rating of insufficient indicated that no or very limited evidence was available or the body of evidence had unacceptable deficiencies, precluding reaching a conclusion.

## RESULTS

We included a total of 278 RCTs (see flow diagram in the online supplement); one systematic review of 138 trials (N=47,189) (17) and 24 additional trials (N=6,672) (21, 22, 23, 24, 25, 26, 27, 28, 29, 30, 31, 32, 33, 34, 35, 36, 37, 38, 39, 40, 41, 42, 43, 44) for SGAs versus other SGAs, and one systematic review of 111 trials (N=118,503) (45, 46) and five additional trials (N=1,055) (35, 47, 48, 49, 50) for FGAs versus SGAs. The two systematic reviews also included 33 cohort studies (N =652,505) (17, 45, 46). Table 1 provides an overview of characteristics of the included RCTs. Although most RCTs were 8 to 12 weeks in duration, some studies were longer (3 to 4 years). The typical patient was age 37 (younger in first‐episode studies) and male. Slightly less than 50% (N=137) of the studies were conducted solely in the United States, and close to 70% (N=193) were funded by the pharmaceutical industry. A majority of studies were judged to be of fair quality; key limitations of RCTs were unclear randomization procedures, unclear or lack of blinding of outcome assessors, and incomplete reporting. Older SGAs (clozapine, risperidone, olanzapine, quetiapine, ziprasidone, and aripiprazole) were most frequently studied, with little comparative evidence for the newest drugs (asenapine, brexpiprazole, cariprazine, iloperidone, lurasidone, paliperidone, and long‐acting injection [LAI] formulations of aripiprazole and pal‐iperidone). Detailed descriptions of studies and systematic reviews can be found in the full report 10). Among the included trials was the Clinical Antipsychotic Trials of Intervention Effectiveness (CATIE) schizophrenia study (51, 52, 53, 54, 55), a large, good‐quality, federally funded effectiveness trial with three phases, examining four SGAs (clozapine, risperidone, olanza‐pine, and ziprasidone) and one FGA (perphenazine), with all‐cause treatment discontinuation as the primary outcome.

**TABLE 1:** Characteristics of RCTs (N=278) included in a review of second‐generation antipsychotics (SGAs) for patients with schizophrenia

Medication	Specific comparisons	N of RCTs	N of patients	Median duration (range)		% female participants	
					Mean age		Study quality
SGA vs. SGA	Most were older SGAs (clozapine, risperidone, olanzapine, quetiapine, ziprasidone, and aripiprazole); few were newest drugs (long‐acting injectables, cariprazine, brexpiprazole)	162^a^	53,861	12 weeks (6 weeks–3 years)	37 years (26 years for first‐episode studies)	22–77	Good, 9%; fair, 73%; poor: 18%
FGA^b^ vs. SGA	Most comparisons involved haloperidol vs. olanzapine and/or risperidone.	116^c^	69,600	8 weeks (1 day–4 years)	37 years	25–42	Good, 0%; fair, 63%; poor, 37%

a 138 randomized controlled trials (RCTs) were included in a good‐quality systematic review, and 24 additional RCTs were identified.

b FGA, first‐generation antipsychotic.

c 111 RCTs were included in a good‐quality systematic review, and five additional RCTs were identified.

Table 2 presents a summary of key findings and the strength of evidence according to drug comparison and outcome. Some harms outcomes (diabetes, weight gain, tardive dyskinesia, extrapyramidal symptoms, and changes in sexual function) were not addressed in the review of FGAs versus SGAs. No body of evidence in these areas met criteria for high‐strength evidence, primarily because of limitations of the individual studies, lack of precise estimates, and inconsistencies across studies (see online supplement). Detailed tables of individual study characteristics, results and quality assessments, and domain‐based ratings of the strength of the bodies of evidence are available in the full report (10). Estimates of comparative risk are reported below only when they are statistically significant.

**TABLE 2:** Summary of key findings and strength of evidence in a review of second‐generation antipsychotics (SGAs), by outcome^a^

Outcome	Moderate strength of evidence	Low strength of evidence
Improved social function		Risperidone LAI significantly better than quetiapine in social function over 24 months. No difference between paliperidone palmitate LAI (monthly) and risperidone LAI (every 2 weeks).
Improved occupational function		No significant differences between risperidone, olanzapine, quetiapine, or ziprasidone at 18 months (CATIE).
Improved global functioning		Global functioning did not differ between olanzapine and either risperidone or quetiapine.
Improved quality of life	Olanzapine did not significantly differ from risperidone, ziprasidone, haloperidol, or perphenazine. Perphenazine did not significantly differ from quetiapine, risperidone, or ziprasidone.	Olanzapine and risperidone did not significantly differ from quetiapine. Risperidone LAI did not significantly differ from quetiapine. Oral aripiprazole did not significantly differ from aripiprazole monthly LAI.
Response	Risperidone did not significantly differ from haloperidol	Response was significantly more likely with olanzapine and risperidone than quetiapine and with olanzapine than with haloperidol. Haloperidol did not significantly differ from aripiprazole, quetiapine, or ziprasidone.
Mortality		No difference between asenapine and olanzapine, quetiapine and risperidone, paliperidone palmitate LAI (monthly) and risperidone, LAI risperidone and olanzapine or quetiapine, or olanzapine and quetiapine (including cardiovascular mortality).
Self‐harm	Clozapine was superior to olanzapine in preventing significant suicide attempts or hospitalization to prevent suicide in high‐risk patients	Clozapine was associated with lower risk of suicide or suicide attempts than were olanzapine, quetiapine, or ziprasidone.
Improved total scale scores	Olanzapine and risperidone improved symptoms more than haloperidol	Clozapine improved symptoms more than the other SGAs, except for olanzapine. Olanzapine and risperidone improved symptoms more than most other SGAs (except for each other and for paliperidone). Paliperidone improved symptoms more than lurasidone and iloperidone did. In patients with treatment‐resistant disorders, olanzapine improved symptoms more than quetiapine.
Overall adverse events	Overall incidence of adverse events did not differ between olanzapine and asenapine. Haloperidol had greater risk of any adverse event than did aripiprazole, risperidone, and ziprasidone.	No significant differences were found between Quetiapine ER vs. quetiapine and risperidone; risperidone vs. clozapine and aripiprazole; olanzapine vs. paliperidone; risperidone LAI vs. paliperidone and paliperidone palmitate monthly LAI; and aripiprazole vs. aripiprazole monthly LAI.
Withdrawal due to adverse events	Haloperidol had greater risk of withdrawals due to adverse event than aripiprazole, olanzapine, risperidone, or ziprasidone.	Based on a network meta‐analysis of 90 trials, risperidone LAI had significantly lower risk than clozapine, lurasidone, quetiapine ER, risperidone, and ziprasidone. Olanzapine had lower risk than clozapine, lurasidone, quetiapine, risperidone, and ziprasidone. Aripiprazole had lower risk than clozapine and ziprasidone. Cariprazine and iloperidone had lower risk than clozapine.

a No interventions met high strength of evidence criteria for any outcome (see online supplement for details of strength of evidence ratings and effect sizes). CATIE, Clinical Antipsychotic Trials of Intervention Effectiveness; ER, extended release; IR, immediate release; LAI, long‐acting injectable.

### Function

#### SGA versus SGA

No significant differences in social junction outcomes, as assessed by a variety of measures, were found between the older oral SGAs, between paliperidone palmitate monthly LAI and risperidone biweekly LAI, or between risperidone and cariprazine (17, 28). Social functioning was better with risperidone LAI than with quetiapine, as measured by the Social and Occupational Functioning Assessment Scale at 6 to 18 months (one RCT, between‐group differences 3.4 to 5.5 on a 100‐point scale) (56). CATIE phase 1 found no significant differences in rates of employment among patients taking risperidone, olanzapine, quetiapine, perphenazine, and zipra‐sidone at 18 months. Global functioning did not differ (as measured by the Global Assessment of Functioning [GAF] scale) among patients taking olanzapine and risperidone (four cohort studies) or quetiapine (two RCTs) (17).

#### FGA versus SGA

In studies comparing FGAs and SGAs, outcomes related to function were rarely reported, with no significant differences reported for any measure of function (45). This evidence included single studies comparing the effects of haloperidol with olanzapine, quetiapine, or ziprasidone on patients' GAF scores, single studies comparing the use of perphenazine with olanzapine, quetiapine, risperidone, or ziprasidone on the proportion of patients with paid employment, and a trial of the effects of haloperidol versus risperidone on patients' economic independence.

### Quality of Life

#### SGA versus SGA

Although there were small improvements from baseline, quality of life did not differ among patients taking older SGAs (clozapine, risperidone oral and LAI, olanzapine, quetiapine, and ziprasidone). Olanzapine did not significantly differ from risperidone (two RCTs), ziprasidone (two RCTs), or quetiapine (one RCT), and risperidone did not differ from quetiapine or ziprasidone (one RCT each) at 12 months, as assessed by patient results on the HeinrichCarpenter Quality of Life Scale. Risperidone LAI did not differ from quetiapine on patient results on the Short Form Health Survey or the Schizophrenia Quality of Life Scale–Revision 4 at 24 months (one RCT) (17).

#### FGA versus SGA

The evidence comparing FGAs and SGAs did not support a differential effect in quality of life, as determined by various measures. Two trials reported inconsistent findings between haloperidol and ziprasidone; five RCTs reported no differences between haloperidol and olanzapine; and one RCT each found no differences between perphenazine and olanzapine, quetiapine, risperidone, or ziprasidone (45).

### Response, Improvement in Symptoms, and Relapse

#### SGA versus SGA

Response to treatment was significantly more likely with olanzapine (five RCTs, odds ratio [OR]=1.71, 95% confidence interval [CI]=1.11 to 2.68) and risperidone (nine RCTs, OR=1.41, 95% CI=1.01 to 2.00) than with quetiapine, according to a network meta‐analysis of head‐to‐head RCTs (N=12,536) (see online supplement) (17). Response ranged from 20% to 80% in individual study arms. A metaregression examining study duration, dose level, treatment resistance, or first‐episode status, and response definition did not identify any predictors of response.

Clozapine was found to improve symptoms (as assessed by scores on the PANSS or Brief Psychiatric Rating Scale) significantly more than the other SGAs (standardized mean differences [SMDs]=−0.32 to −0.55) (57). Olanzapine and risperidone were similar to each other, but demonstrated greater improvements than the other SGAs, except for paliperidone (SMDs=−0.13 to −0.26) (57). Paliperidone was found to improve symptoms more than lurasidone and iloperidone (both SMDs=−0.17).

The evidence on relapse suffers from lack of blinding, high attrition, and lack of a consistent definition of relapse (17). Evidence on the comparison of olanzapine with risperidone and quetiapine was inconsistent, and conclusions could not be drawn. Risperidone LAI resulted in lower relapse rates than oral risperidone (5% to 18% versus 33% to 50%; p<0.010) or quetiapine (16.5% versus 31.3%; p<0.001) at 1 year (one RCT each). In the few studies available, no differences were found in comparisons of risperidone and quetiapine to each other or to clozapine and lurasidone. No differences in relapse rates were found for comparisons of lurasidone and quetiapine extended release (ER) or risperidone, risperidone and quetiapine ER, olanzapine and aripiprazole, or aripi‐prazole LAI or risperidone LAI.

For patients with first‐episode psychosis, no significant differences were found between oral olanzapine, quetiapine, risperidone, ziprasidone, aripiprazole, or paliperidone (17 RCTs) in response, remission, or symptom improvement, regardless of study duration, specific drugs, age or gender, history of cannabis use disorder, or treatment blinding, but aripiprazole resulted in more weight gain than ziprasi‐done (17).

#### FGA versus SGA

Compared with haloperidol, olanzapine use was associated with greater response (14 RCTs, relative risk [RR]=0.86, 95% CI=0.78 to 0.96) and remission (three RCTs; RR=0.64, 95% CI=0.45 to 0.94) (45). There was no difference in response between haloperidol and aripiprazole, quetia‐pine, risperidone, and ziprasidone and no difference in remission versus ziprasidone (45).

Reductions in symptoms were greater with older SGAs than with haloperidol. Compared with haloperidol, total PANSS scores were better with olanzapine (15 RCTs, mean difference [MD]=2.31, 95% CI=0.44 to 4.18) and risperidone (21 RCTs, MD=3.24, 95% CI=1.62 to 4.86).

For patients with a first episode of schizophrenia and/or psychosis, the evidence showed no significant differences between FGAs and SGAs. Among patients with treatment‐resistant disorders, response was significantly better with ziprasidone (one RCT, RR=1.54, 95% CI=1.19 to 2.00) than with FGAs.

### Discontinuation of Treatment

#### SGA versus SGA

Results of a network analysis (111 studies, N=32,096) found that olanzapine and clozapine had significantly lower treatment discontinuation rates than asenapine, cariprazine, iloperidone, lurasidone, olanzapine LAI, que‐tiapine, risperidone, and ziprasidone (odds ratios [ORs] ranged from 0.42 for clozapine versus iloperidone to 0.69 for clozapine versus risperidone) (17). Clozapine also resulted in lower risk of treatment discontinuation than paliperidone palmitate monthly LAI (OR=0.56, 95% CI=0.33 to 0.92), and olanzapine resulted in lower risk than paliperidone (OR=0.67, 95% CI=0.50 to 0.89). Quetiapine ER resulted in lower risk of treatment discontinuation than iloperidone (OR=0.28, 95% CI=0.09 to 0.84), olanzapine LAI (OR=0.31, 95% CI=0.09 to 0.99), or quetiapine (OR=0.35, 95% CI=0.12 to 0.88), and both risperidone and aripiprazole had lower risk than iloperidone (OR=0.62, 95% CI=0.43 to 0.90; OR=0.61, 95% CI=0.40 to 0.94, respectively) or quetiapine (OR=0.77, 95% CI=0.66 to 0.91; OR=0.76, 95% CI=0.61 to 0.95, respectively). Both risperidone (OR=0.62, 95% CI=0.43 to 0.90) and aripiprazole monthly LAI (OR=0.52, 95% CI=0.28 to 0.98) had lower risk of treatment discontinuation than iloperidone. A meta‐regression analysis examining study duration, dose level, and either treatment‐resistant or first‐episode status resulted in no significant findings.

Time to discontinuation was 2 to 4 months longer with olanzapine compared with quetiapine (17 studies), risperidone (31 studies), and ziprasidone (10 studies), on the basis of trial and observational evidence. Time to discontinuation may be longer with clozapine (7.2 to 7.8 months longer) than with olanzapine, risperidone, or quetiapine, as assessed by phase 2E of the CATIE trial (51).

Results of a network meta‐analysis (90 RCTs, N=29,678) revealed significantly lower withdrawals because of adverse events with risperidone LAI than with clozapine (OR=0.27, 95% CI=0.10 to 0.71), lurasidone (OR=0.39, 95% CI=0.18 to 0.84), quetiapine (OR=0.43, 95% CI=0.22 to 0.81), risperidone (OR=0.50, 95% CI=0.25 to 0.99), and ziprasidone (OR=0.40, 0.20 to 0.82). Olanzapine had lower risk than clozapine (OR=0.39, 95% CI=0.19 to 0.79), lurasidone (OR=0.57, 95% CI=0.34 to 0.94), quetiapine (OR=0.62, 95% CI=0.44 to 0.87), risperidone (OR=0.72, 95% CI=0.55 to 0.96), and ziprasidone (OR=0.58, 95% CI=0.41 to 0.82). Aripiprazole had lower incidence of withdrawals than ziprasidone (OR=0.64, 95% CI=0.44 to 0.94) and clozapine (OR=0.43, 0.21 to 0.88). Cariprazine (OR=0.40, 95% CI=0.17 to 0.95) and iloperidone (OR=0.34, 95% CI=0.13 to 0.91) had lower risk of withdrawal than clozapine. Our meta‐regression analysis resulted in no significant findings.

#### FGA versus SGA

All‐cause discontinuation of treatment was not reported in the systematic review of FGAs versus SGAs (45). However, withdrawals due to adverse events were significantly higher with haloperidol use compared with aripi‐prazole (eight RCTs, RR=1.25), olanzapine (24 RCTs, RR=1.89), risperidone (25 RCTs, RR=1.32), and ziprasidone (27 RCTs, RR=1.68) (35, 45, 47, 48, 49). There were no differences in withdrawal due to adverse events in comparisons of haloperidol and clozapine (five RCTs) or quetiapine (10 RCTs). On the basis of single studies for each comparison, no significant differences were found in comparisons of haloperidol to asenapine; fluphenazine to olanzapine or quetiapine; and perphenazine to aripiprazole, olanzapine, quetiapine, risper‐idone, or ziprasidone.

### Mortality and Self‐Harm

#### SGA versus SGA

In 10 RCTs, incidence of all‐cause mortality was low, and did not differ between the SGAs over 4 to 24 months; evidence was not available for the newest SGAs (10, 21, 33, 58, 59, 60, 61, 62, 63, 64, 65). In a good quality RCT, among patients at high risk for suicide, clozapine was found to be superior to olanzapine in preventing substantial suicide attempts or hospitalization to prevent suicide (hazard ratio [HR]=0.76, 95% CI=0.58 to 0.97) and in CGI‐Severity‐Suicidality Scale ratings (HR=0.78, 95% CI=0.61 to 0.99) (66). Two observational studies confirmed these findings in broader populations (67, 68).

#### FGA versus SGA

Mortality was not reported in the trials included in the systematic review comparing FGAs and SGAs, and the majority of trials excluded people at risk of suicide (45). No differences were found in risk of suicide outcomes between perphenazine or haloperidol and olanzapine (45) or between LAI haloperidol and LAI paliperidone, as evidenced by a single trial per comparison (50).

### Diabetes Mellitus and Weight Gain

The evidence directly comparing SGAs on incidence of diabetes mellitus, ketoacidosis, and weight gain was limited and did not adequately control for confounding factors (17). Olanzapine was associated with increased risk of new‐onset diabetes compared with risperidone (six cohort studies, OR=1.16, 95% CI=1.03 to 1.31), but differences were not consistently found among other older SGAs (17). Evidence on the incidence diabetic ketoacidosis with olan‐zapine, risperidone, and quetiapine was inconsistent (two studies).

The risk of clinically important weight gain (≥7% increase) was greater with olanzapine than with aripiprazole, asenapine, clozapine, quetiapine, risperidone, or ziprasidone, with relative risks from 1.81 with risperidone to 5.76 with ziprasidone (55 RCTs) (17). Single studies found greater risk of weight gain with risperidone compared with aripiprazole (OR=4.74,95% CI=1.55 to 14.47) or cariprazine (RR=1.98,95% CI=1.03 to 3.80) and with aripiprazole compared with ziprasidone (OR=2.72, 95% CI=1.22 to 6.08) (10, 69). Single studies found no differences in weight change between olanzapine and olanzapine ER, olanzapine oral dissolving tablet, and paliperidone palmitate injection, or between aripiprazole and paliperidone or quetiapine.

### Overall Adverse Event Incidence, Tardive Dyskinesia, Extrapyramidal Symptoms, and Sexual Function

There were no significant differences between the SGAs in the proportions of patients reporting any adverse event, as assessed by 72 RCTs and 31 drug comparisons (17). Observational evidence suggested that compared with olan‐zapine, risperidone significantly increased the risk of new‐onset tardive dyskinesia (OR=1.70, 95% CI=1.35 to 2.14), but not compared with clozapine or quetiapine (17). Rates of new‐onset tardive dyskinesia were 3% with risperidone and 1%‐2% for the other medications. The rates of patients experiencing extrapyramidal side effects (prevalent or incident) and severe symptoms mostly did not differ among the drugs, although use of anticholinergic medications was lower with quetiapine than with olanzapine, risperidone, or ziprasidone.

Evidence on sexual function revealed inconsistent findings; studies had small samples sizes and often lacked explicit methodology to measure symptoms (17). A study comparing risperidone and quetiapine ER (N=798) found that significantly more men reported adverse sexual effects with ris‐peridone at 6 months (13% versus 6%; p<0.050), but the difference was not significant at 12 months. Four very small trials of risperidone compared with quetiapine were inconclusive, and individual trials found no significant differences in results between olanzapine and paliperidone, risperidone, or ziprasidone or between risperidone and paliperidone or aripiprazole.

Overall adverse event rates (incidence of any adverse event) were greater with haloperidol than with aripiprazole (three RCTs, RR=1.11; 95% CI=1.06 to 1.17), risperidone (eight RCTs, RR=1.20, 95% CI=1.01 to 1.42), and ziprasidone (six RCTs, RR=1.13, 95% CI=1.03 to 1.23). Withdrawals due to adverse events were significantly higher with haloperidol than with SGAs: compared with aripiprazole (eight RCTs, RR=1.25, 95% CI=1.07 to 1.47), olanzapine (24 RCTs, RR=1.89; 95% CI=1.57 to 2.27), risperidone (25 RCTs, RR=1.32; 95% CI=1.09 to 1.60), and ziprasidone (seven RCTs, RR=1.68, 95% CI=1.26 to 2.23).

### New Evidence

Since the finalization of the AHRQ report, we identified two new, small RCTs that focused on long‐acting injectable formulations: a 12‐month study conducted in Italy of patients with co‐occurring substance use disorder (aripiprazole LAI versus paliperidone LAI) and a 13‐week study conducted in China of patients with first‐episode schizophrenia (paliperidone LAI versus oral olanzapine) (70, 71). These studies showed no differences in symptom‐based outcomes or in weight gain for the 13‐week study of first‐episode patients (71). Results of the study of patients experiencing comorbid substance use disorder and psychosis (89% with schizophrenia, 11% with bipolar disorder) (70) revealed better improvement on cravings and some quality of life measures with aripiprazole; however, baseline differences between the groups suggest that caution must be used in interpreting these findings.

## DISCUSSION

We evaluated the evidence on antipsychotic medications for schizophrenia, comparing drugs with each other, from 278 RCTs among 173,419 patients. Trials were mostly of fair quality (i.e., moderate risk of bias), and bodies of evidence for specific outcomes and intervention pairs were primarily of low and moderate strength, meaning we had low‐to‐moderate confidence that the findings were stable (i.e., are unlikely to change significantly with additional evidence). The majority of evidence from head‐to‐head drug trials was about older SGAs, with sparse data on SGAs approved in the past 10 years (asenapine, lurasidone, iloperidone, cariprazine, brexpipra‐zole) and recent long‐acting injection formulations of aripi‐prazole and paliperidone. Older SGAs were similar in measures of function, quality of life, mortality, and overall and/or any adverse events, except that patients taking risperidone LAI demonstrated better social function than patients taking que‐tiapine. Symptoms improved more with olanzapine and ris‐peridone than with asenapine, quetiapine, and ziprasidone and more with paliperidone than lurasidone and iloperidone; all were superior to placebo. Risperidone LAI and olanzapine resulted in less withdrawal due to adverse events. Compared with olanzapine and risperidone, haloperidol, the most studied FGA, resulted in similar improvement in symptoms, symptom response, and remission but greater incidence of adverse event outcomes, such as tardive dyskinesia.

These findings are consistent with findings of prior systematic reviews that have made comparisons among the SGAs and between SGAs and FGAs, although our findings differed to some extent from previous reviews because we considered outcomes prioritized with input from technical experts, incorporated newer evidence and the most recently approved drugs, and included three updated network metaanalyses (57, 72, 73, 74, 75, 76). For example, in comparing SGAs, our network meta‐analyses of patient response, withdrawal due to adverse events, and all‐cause treatment discontinuation incorporated evidence on brexpiprazole and cariprazine, the two most recently approved oral drugs, and all of the long‐acting injection SGAs, whereas previously published network meta‐analyses were limited to older oral drugs, did not control for important potential effect modifiers, and included drugs not approved in the United States (57, 72, 74, 76, 77, 78, 79). Our review is consistent with other reviews of the older SGAs in that clozapine, risperidone, and olanzapine showed the most consistent evidence of superiority for specific outcomes (e.g., symptom improvement, response, self‐harm, all‐cause treatment discontinuations, and time to discontinuation) or populations (those with a first episode or treatment‐resistant disorders) (75, 78, 80, 81, 82). Some of our other findings are new, such as the finding that risperidone LAI and olan‐zapine resulted in significantly fewer withdrawals caused by adverse events than most other SGAs. Our findings on FGAs versus SGAs are mainly consistent with those of a prior review (45), which concluded that there were few clinically important differences in effectiveness outcomes and that the evidence on "patient‐important" outcomes and adverse events was not well studied. New findings include moderate‐strength evidence of specific SGAs resulting in better symptom improvement (olanzapine and risperidone) and lower rates of overall adverse events (aripiprazole) and withdrawal due to adverse events (aripiprazole, olanzapine, risperidone, and ziprasidone) than haloperidol.

Potential limitations of our review included decisions that were made regarding eligibility criteria for the AHRQ report. Because the scope of the overall report was broad, incorporating both drug and psychosocial interventions, with a strict budget and time line, some decisions were made to improve efficiency. These constraints included focusing on longer‐term outcomes, limiting studies to those of 12 weeks or longer, including only studies that directly compared the drugs, and incorporating preexisting systematic reviews. These decisions were made after consultation with an expert panel, AHRQ, and representatives of the APA guideline group, and the protocol was posted publicly. The duration threshold reflected the clinical consideration that treatments are typically given for extended periods and that results found in short‐term trials are not always sustained. Limiting the review to head‐to‐head trials also reflected our experience that the findings of indirect comparisons using placebo‐controlled trials can be misleading (83, 84). This finding may be due to the narrower inclusion criteria used by most trials designed for drug approval, and, importantly with antipsychotics, some drugs had doses way above those used clinically today. There may be concern that our use of two preexisting systematic reviews may have resulted in double‐counting data from some studies. Each of the reviews focused on disparate comparisons, such that data for comparisons were not included twice. For example, if an RCT had three arms, two with SGAs and one with an FGA, data for the SGA comparison were included in one of the reviews, whereas data from the FGA versus SGA comparison were included only in the other review.

The findings of this review are applicable to adults (mean age 25 to 50) with mainly moderate and moderate‐to‐severe disease. There was fairly robust evidence on first‐episode patients, but less on patients with treatment‐resistant disorders, and the evidence was not clearly applicable to adolescents, older adults, or patients with multiple comorbid conditions. For the SGAs versus each other, there was evidence for all of the prioritized outcomes; however, the majority of the evidence on effectiveness (long‐term health outcomes) was limited to the older drugs. For FGAs versus SGAs, the outcomes were more limited, with little good‐quality evidence on effectiveness outcomes, such as function, quality of life, self‐harm, and mortality. There was little evidence on long‐term follow‐up (greater than 2 years). For SGAs compared with each other, the evidence applied only to outpatients, while almost half the studies of FGAs versus SGAs were conducted among inpatients.

Although study quality was not a key limitation, funding source may have been, because more than 80% of the studies were funded by the manufacturer of one of the drugs in the trial. Other limitations included variability in patient characteristics, which were often poorly reported, and variability in the specific outcomes selected for study and how they were measured or reported. Consensus on which outcomes are most important and how to best measure them is needed; for example, the primary outcome measure in the CATIE trials (time to discontinuation of drug), which was selected for well‐publicized reasons, was not prioritized by our expert panel. Although many of the older studies lacked general‐izability to the real‐life practice setting, because either the doses used were higher or lower than those used in practice today or the dose comparisons were unfair (e.g., a low dose of one drug versus a high dose of a comparator drug), our analysis of these issues as part of our network analyses indicated that more recent studies have fewer issues with dosing. In comparing drugs, an important limitation was that the typical dosing for some drugs has changed over time, with lower doses used in more recent studies of several older drugs. Also, the prior treatment experience of patients enrolled varied over time (fewer patients had prior FGA exposure or resistance in more recent studies). Evidence on the subgroups was limited by the small sample sizes, and most analyses were post hoc rather than either preplanned analyses or trials designed to address these subgroups as their primary objective. Although we limited our analyses to direct, head‐to‐head comparisons of drugs, the inclusion of indirect (e.g., placebo‐controlled trial) evidence may have led to erroneous conclusions (85, 86). The specific characteristics of enrolled patients, dosing, or other treatment considerations may have played a role in the results, particularly when the indirect evidence was based on studies conducted for marketing approval of a drug. An example of this situation may be seen with clozapine, which was typically found to be superior to all other antipsychotics among patients with treatment‐resistant disease. In contrast, we found that olanzapine and risperidone may also be useful, and we did not find clozapine to be highly superior. In related prior work, we found that older placebo‐controlled trials of clozapine enrolled patients with very severe symptoms, in whom a larger margin of change was likely to be seen with an effective treatment, and that clozapine dosing was much higher than is acceptable today (87).

Our findings are mainly compatible with other recent systematic reviews and meta‐analyses, which have varied in scope and focus (88, 89, 90). For example, a recent large network meta‐analysis (88) covered many of the same SGAs and outcomes as our review but excluded studies of patients with treatment‐resistant disease, a first episode, or with predominant negative symptoms. Importantly, many of the drugs included in the network meta‐analysis (88) are not available in the United States at this time or were identified as a drug not currently used to treat schizophrenia (54% of the drugs in the network). Inclusion of such a large amount of data that is not relevant to the current U.S. setting would influence the results. Other fairly recent reviews have focused on specific subpopulations of patients with schizophrenia (e.g., first episode or long duration studies only) and also have included drugs not available in the United States (89, 90). All of these reviews excluded the newer long‐acting injectable drugs, which were of key interest to inform APA guidelines. These scope issues are decisions that were made early in the review process; our review was intentionally broad in population and outcome criteria and specific to the needs of the APA for drug interventions studied. As required to perform review work for AHRQ, none of our review team had conflicts of interest to declare, whereas this was not true for these other recent reviews.

On the basis of the limitations identified here, several research recommendations can be made. Future studies should involve multiple newer SGA drugs (approved in the past 10 years); include comparable dosing with the most appropriate dosing titration methods for all drugs included; measure key health outcomes (including harms); have study durations of 3 to 5 years of follow‐up; incorporate the concept of recovery into study designs (i.e., inclusion of recovery as a primary outcome, with an a priori definition and criteria), with testing of duration of effect and discontinuation of drug treatment following remission; enroll patients who reflect real populations (e.g., older patients, multiple comorbidities, concomitant medications, severe disease); and prespecify subgroup analyses (e.g., women and racial‐ethnic groups).

## CONCLUSIONS

Most of the comparative evidence has described older SGAs (clozapine, olanzapine, risperidone, quetiapine, and zipra‐sidone), with some evidence describing paliperidone and aripiprazole and newer long‐acting injectable formulations, and very little addressing newer SGAs approved in the past 10 years (asenapine, brexpiprazole, cariprazine, iloperidone, and lurasidone). Although none were superior on multiple high‐priority outcomes, among the SGAs, clozapine, olan‐zapine, and risperidone oral and LAI showed superior performance on more outcomes, and quetiapine and ziprasidone were not better on any outcome. On the basis of limited evidence, newer SGAs were not found to be superior to older SGAs. Compared with haloperidol, olanzapine, risperidone, ziprasidone, and aripiprazole performed similarly on benefit outcomes and resulted in lower incidence of adverse events or withdrawals.

## Supporting information

Supplementary material
