# Supplementary material for: Updating the Comparative Evidence on Second‐Generation Antipsychotic Use With Schizophrenia
Source: Psychiatr Res Clin Pract. 2020 Oct 16;2(2):76–87. doi: 10.1176/appi.prcp.20200004 (PMC9175869; doi:10.1176/appi.prcp.20200004)
Supplement: Supplementary file 1 — Supplementary material [file RCP2-2-76-s001.pdf]

Literature flow diagram

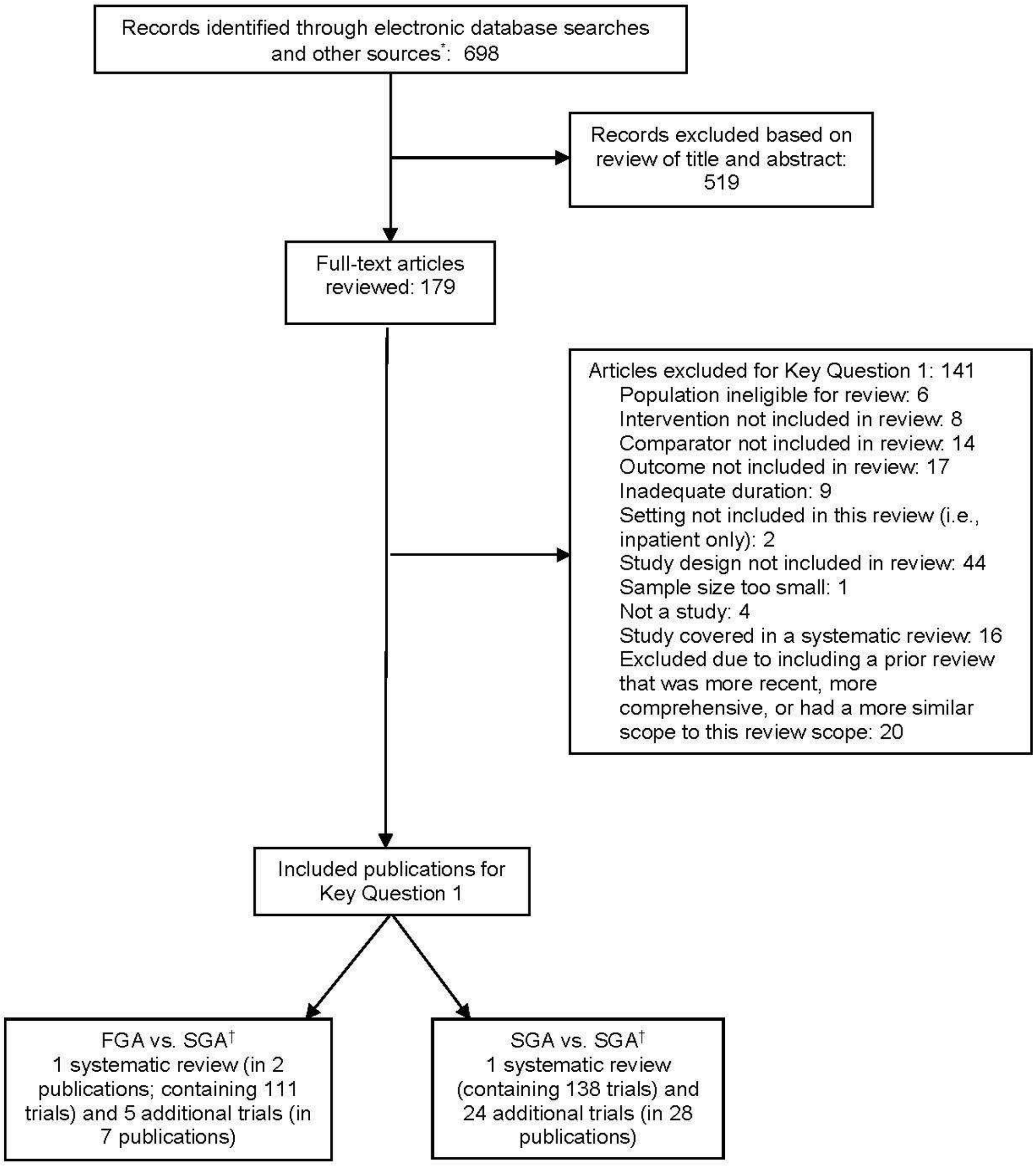

FGA = first-generation antipsychotic; SGA = second-generation antipsychotic
[*] Other sources include prior reports, references lists, referrals from experts, and grey literature.
[†] Some studies were included for both FGA vs. SGA and SGA vs. SGA sections.

Network Meta-analysis results for Response: SGA versus SGA (odds ratio, 95% confidence interval)

**Aripiprazole**	1.05 (0.35-3.02)	0.97 (0.57-1.68)	0.57 (0.20-1.63)	1.16 (0.56-2.58)	0.70 (0.35-1.45)	0.67 (0.45-1.02)	1.11 (0.65-2.00)	1.15 (0.77-1.78)	0.81 (0.58-1.16)	0.98 (0.59-1.58)
0.96 (0.33-2.86)	**Aripiprazole LAI-1**	0.93 (0.28-3.13)	0.55 (0.13-2.44)	1.12 (0.30-4.26)	0.67 (0.25-1.89)	0.65 (0.18-2.26)	1.06 (0.32-3.78)	1.08 (0.35-3.57)	0.78 (0.25-2.52)	0.93 (0.28-3.16)
1.03 (0.59-1.76)	1.08 (0.32-3.53)	**Asenapine**	0.59 (0.18-1.81)	1.20 (0.54-2.82)	0.72 (0.33-1.64)	0.69 (0.41-1.20)	1.15 (0.57-2.32)	1.18 (0.69-2.09)	0.83 (0.49-1.43)	1.01 (0.54-1.86)
1.75 (0.61-5.05)	1.83 (0.41-7.68)	1.69 (0.55-5.44)	**Brexpiprazole**	2.03 (0.62-7.08)	1.23 (0.33-4.52)	1.18 (0.36-3.90)	1.94 (0.58-6.64)	1.99 (0.67-6.25)	1.43 (0.46-4.46)	1.70 (0.53-5.51)
0.86 (0.39-1.79)	0.90 (0.24-3.31)	0.83 (0.36-1.87)	0.49 (0.14-1.63)	**Cariprazine**	0.59 (0.23-1.56)	0.58 (0.25-1.26)	0.95 (0.39-2.36)	0.98 (0.48-2.04)	0.70 (0.33-1.44)	0.84 (0.36-1.87)
1.43 (0.69-2.88)	1.50 (0.53-4.06)	1.38 (0.61-3.05)	0.82 (0.22-3.00)	1.69 (0.65-4.38)	**Clozapine**	0.96 (0.46-1.96)	1.58 (0.69-3.71)	1.63 (0.84-3.36)	1.16 (0.61-2.24)	1.39 (0.63-2.94)
1.49 (0.98-2.22)	1.54 (0.45-5.48)	1.44 (0.83-2.47)	0.85 (0.26-2.79)	1.74 (0.79-3.94)	1.04 (0.51-2.19)	**Olanzapine**	1.65 (0.95-2.95)	***1.71 (1.11-2.68)***	1.21 (0.94-1.59)	1.45 (0.94-2.25)
0.90 (0.50-1.54)	0.94 (0.26-3.12)	0.87 (0.43-1.75)	0.52 (0.15-1.74)	1.05 (0.42-2.56)	0.63 (0.27-1.45)	0.61 (0.34-1.05)	**Paliperidone**	1.03 (0.55-1.94)	0.73 (0.42-1.27)	0.88 (0.44-1.71)
0.87 (0.56-1.29)	0.92 (0.28-2.85)	0.85 (0.48-1.46)	0.50 (0.16-1.49)	1.02 (0.49-2.09)	0.61 (0.30-1.19)	***0.59 (0.37-0.90)***	0.97 (0.52-1.81)	**Quetiapine**	***0.71 (0.50-0.99)***	0.85 (0.50-1.39)
1.23 (0.86-1.71)	1.29 (0.40-4.08)	1.20 (0.70-2.02)	0.70 (0.22-2.16)	1.43 (0.69-3.00)	0.86 (0.45-1.65)	0.83 (0.63-1.07)	1.36 (0.79-2.40)	***1.41 (1.01-2.00)***	**Risperidone**	1.20 (0.78-1.82)
1.03 (0.63-1.70)	1.08 (0.32-3.59)	0.99 (0.54-1.85)	0.59 (0.18-1.90)	1.19 (0.53-2.81)	0.72 (0.34-1.58)	0.69 (0.44-1.07)	1.14 (0.59-2.27)	1.18 (0.72-2.01)	0.84 (0.55-1.28)	**Ziprasizone**

Comparisons between treatments should be read from left to right and the estimate in each cell represents comparison between the row-defining treatment and the column-defining treatment. Odds ratios (ORs) higher than 1 favor the column-defining treatment, meaning less withdrawal in the column-defining treatment; and ORs less than 1 favor the row-defining treatment, meaning less withdrawal in the row-defining treatment. For example, the OR for comparing QUE (row-defining treatment) vs. RIS (column-defining treatment) is 0.71 (95% CrI 0.50 to 0.99), which means that QUE has lower odds of response and the comparison favors RIS, which has greater response. Each comparison is represented twice in the table, for example, for two treatments A and B, once with A versus B and once with B versus A (i.e. the reciprocal OR). For the example above, the OR for comparing RIS (now the row-defining treatment) vs. QUE (now the column-defining treatment) is 1.41 (95% CI 1.01 to 2.00), which means that RIS has a higher odds of response and again, it means that the comparison favors RIS, which has greater response. Significant results are in bold. LAI = long-acting injection; 1 = once monthly-

Strength of Evidence Ratings

Table 1. Strength of evidence: second-generation antipsychotic versus second-generation antipsychotic

Outcome	Comparators	Number of Studies Number of Subjects	Study Limitations	Consistency	: Directness	Precision	Magnitude of Effect: Summary Effect Size (95% CI)	Strength of Evidence (High, Moderate, Low, Insufficient)
**Social Functioning**	Olanzapine, risperidone, quetiapine immediate-release	1 SR[1] (2 RCTs; N =358;) (1 observational study; N = 9028)	Moderate<br><br>Observational evidence: Moderate	Inconsistent<br><br>Observational evidence: Unknown	Direct<br><br>Observational evidence: Direct	Imprecise<br><br>Observa-tional evidence: Precise	Inconclusive:<br>RCT 1: no significant differences on RFS or the SAS-SMI<br>RCT 2: Change on SFS greater with App H olanzapine (+7.75) than risperidone (-0.92; P=0.0028) (Socially active: OR 1.27, 95% CI 1.05 to 1.54 olanzapine 84.6% vs. risperidone 82.4%).	Insufficient
Social Functioning	Paliperidone monthly LAI vs. risperidone q 2 wks LAI	1 SR[1] (2 RCTs; N = 452)	Moderate	Inconsistent	Direct	Precise	No statistically significant differences in PSP scale: mean change from baseline 16.8 paliperidone and 18.6 risperidone; (LSM difference 0.5, 95% CI -2.14 to 3.12)	Low
Social Functioning	Paliperidone extended release vs. olanzapine	1 Meta-analysis of selected studies[2]	High	Unknown	Direct	Precise	No significant difference in PSP scale (change 7.8 to 12.2 in paliperidone dose u vs. 8.7 in olanzapine group).	Insufficient

Outcome	Comparators	Number of Studies Number of Subjects	Study Limitations	Consistency	: Directness	Precision	Magnitude of Effect: Summary Effect Size (95% CI)	Strength of Evidence (High, Moderate, Low, Insufficient)
Social Functioning	Risperidone LAI vs. Quetiapine immediate release	1 RCT[3] (N = 666)	Moderate	Unknown	Direct	Precise	Risperidone LAI resulted in greater improvements in SOFA at 6 months (6.1 vs. 2.7; P = 0.02), 12 months (9.5 vs. 6.1; P = 0.009), and endpoint (6.6 vs. 1.1; P < 0.0001).	Low
**Employment Outcomes**	Older SGAs (olanzapine, risperidone, quetiapine, ziprasidone)	1 SR[1] (2 RCTs, 3 observational N = 1,379)	Low<br><br>Observational evidence: Moderate	Inconsistent<br><br>Observational evidence: Consistent	Direct<br><br>Observational evidence: Direct	Imprecise<br><br>Observa-tional evidence: Imprecise	No significant differences in rates of employment (mean 18% in CATIE Phase I)	Low
**Occupation and Residential Status**	Older SGAs (olanzapine, risperidone, quetiapine, ziprasidone)	1 SR[1] (21 RCT, N = 771)	Moderate	Unknown	Direct	Imprecise	Inconclusive:<br>75.5% and 75.3% had stable status, 3.8% and 3.1% improved status (NS)	Insufficient
**Global Functioning (GAF)**	Olanzapine vs. Risperidone	1 SR[1] (4 cohort studies; N = 3211)	High	Inconsistent	Direct	Precise	No difference:<br>Pooled WMD 0.61, 95% CI, -1.78 – 2.99; I^2 = 43%	Low
Global Functioning (GAF)	Olanzapine vs. Quetiapine	1 SR[1] (2 RCTs; N = 363)	Moderate	Consistent	Direct	Imprecise	Pooled WMD 1.14, 95% CI -4.75 to 7.02; Q = 3.99, df = 1; P = 0.045	Low
**Quality of Life**	Olanzapine vs. risperidone	1 SR[1] (2 RCTs; N = 492)	Moderate	Consistent	Direct	Precise	QLS Change = 12 months 0.19 v 0.26 (P = 0.53); 7 months 13.4 vs. 8.8 (P >0.074)	Moderate
Quality of Life	Olanzapine vs. ziprasidone	1 SR[1] (2 RCTs; N = 740)	Moderate	Consistent	Direct	Precise	QLS Change = 12 months 0.19 v 0.26 (P NR); 6-7 months Endpoint 61.3 vs. 58.9 (P = 0.36 using mixed-effect modeling)	Moderate
Quality of Life	Olanzapine vs. quetiapine immediate release	1 SR[1] (1 RCT; N = 227)	Low	Unknown	Direct	Imprecise	QLS Change = 12 months 0.19 v 0.09 (P >0.05)	Low

Outcome	Comparators	Number of Studies Number of Subjects	Study Limitations	Consistency	: Directness	Precision	Magnitude of Effect: Summary Effect Size (95% CI)	Strength of Evidence (High, Moderate, Low, Insufficient)
Quality of Life	Olanzapine vs. asenapine	1 SR[1] (1 RCT; N = 464)	Moderate	Unknown	Direct	Imprecise	QLS Change =12 months 11.7 vs. 11.8 and 11.1 vs. 7.1 (multi-country study reported by hemisphere) P = NS	Insufficient
Quality of Life	Olanzapine vs. clozapine	1 SR[1] (1 RCT; N = 114)	Moderate	Unknown	Direct	Imprecise	SWN: 26 weeks O found non-inferior to C (difference 3.2, 95% CI: 4.2 to 10.5)	Insufficient
Quality of Life	Risperidone vs. ziprasidone	1 SR[1] (N = 154)	Low	Unknown	Direct	Imprecise	QLS Change = 12 months 0.19 v 0.26 (P >0.05)	Low
Quality of Life	Risperidone vs. Quetiapine	1 SR[1] (1 RCT; N = 189)	Low	Unknown	Direct	Imprecise	QLS Change = 12 months 0.26 v 0.26 (P >0.05);	Low
Quality of Life	Quetiapine-ER vs. Risperidone	1 RCT; N = 798[4]	Moderate	Unknown	Direct	Imprecise	SWN 20% Response 12 months: 65% vs. 68%, adjusted difference (-5.7, 95% CI -15.1 to 3.7) but not meet non-inferiority criteria	Insufficient
Quality of Life	Aripiprazole oral vs. aripiprazole LAI (monthly)	1 RCT; N = 724[5]	Moderate	Unknown	Direct	Precise	SF-36 12 months: mental component scores (0.82 vs. 0.38; difference 0.44, 95% CI -1.24 to 2.12) and physical component scores (0.23 vs. -0.27; difference 0.50, 95% CI -1.11 to 2.11)	Low
Quality of Life	Aripiprazole LAI vs. paliperidone palmitate LAI (monthly)	1 RCT; N = 295[6]	Moderate	Unknown	Direct	Imprecise	QLS: 28 weeks change = 7.47 vs. 2.80 (least squares mean difference 4.67, 95% CI 0.32 to 9.02). Meets non-inferiority criteria; not meet MCID	Insufficient

Outcome	Comparators	Number of Studies Number of Subjects	Study Limitations	Consistency	: Directness	Precision	Magnitude of Effect: Summary Effect Size (95% CI)	Strength of Evidence (High, Moderate, Low, Insufficient)
Quality of Life	Risperidone LAI vs. quetiapine	1 RCT; N = 666[3]	Moderate	Unknown	Direct	Precise	SF-12 physical and mental component scores and SQLS-R4 scores improved from baseline in both groups but were not significantly different at endpoint (24 months; SF-12 physical P= 0.09; SF-12 mental and SQLS-R4 P = NR).	Low
**Response**		Network meta-analysis of olanzapine, risperidone, quetiapine IR, aripiprazole, clozapine, ziprasidone> asenapine, paliperidone, aripiprazole LAI monthly, carpipramine, brexpiprazole, lurasidone 46 RCTs (N = 12,536)	Moderate	Consistent	Indirect	Precise	There were 2 statistically significant differences between the drugs; both olanzapine (OR 1.71. 95% CI 1.11,2.68) and risperidone (OR 1.41, 95% CI 1.01,2.00) were significantly more likely to result in response than quetiapine IR.	Low
**Mortality (All-Cause)**	Olanzapine vs. risperidone vs. quetiapine	1 SR[1] (1 retrospective cohort study; N = 48, 595)	Low	Unknown	Direct	Precise	No difference in all-cause mortality between risperidone and olanzapine (HR 1.09, 95% Cl 0.79 to 1.49) or quetiapine (HR 0.75, 95% Cl 0.53 to 1.07).	Low
Mortality (All-Cause)	Clozapine, risperidone, olanzapine and quetiapine vs. no treatment	1 SR[1] (1retrospective cohort study; N = 6, 987)	Low	Unknown	Direct	Imprecise	Clozapine and quetiapine had significantly lower risk of all-cause mortality (adjusted ORs 0.35, 95% CI 0.21 to 0.58 and 0.46, 95% CI 0.30 to 0.72), risperidone and olanzapine not statistically significantly different from control	Insufficient
Mortality (All-Cause)	Asenapine vs. Olanzapine	2 RCTs[7,8]	Moderate	Consistent	Direct	Imprecise	Inconclusive: 0.41% vs. 0.42%, 0% vs. 0%, 0.77% vs. 0.32%; RR 2.49 (95% CI 0.54 to 11.5)	Low

Outcome	Comparators	Number of Studies Number of Subjects	Study Limitations	Consistency	: Directness	Precision	Magnitude of Effect: Summary Effect Size (95% CI)	Strength of Evidence (High, Moderate, Low, Insufficient)
Mortality (All-Cause)	Paliperidone palmitate LAI (monthly) vs. Risperidone LAI	2 RCTs[9,10]	Moderate	Consistent	Direct	Imprecise	Inconclusive: 0.79% vs. 0.27%, 0% vs. 0.45%; RR 1.26 (95% CI 0.21 to 7.49)	Low
Mortality (All-Cause)	Quetiapine vs. Risperidone	2 RCTs[11,12]	Moderate	Consistent	Direct	Imprecise	Inconclusive: 1.17% vs. 0.40% and 0.72% vs. 0%; RR 3.24 (95% CI 0.72 to 14.6)	Low
**Cardio-vascular Mortality**	Olanzapine vs. risperidone vs. quetiapine	1 SR[1] (2 retrospective cohort study; N = 55,582)	Low	Consistent	Direct	Precise	No significant differences between the drugs (HRs 0.99, 95% Cl 0.37 to 2.67 and 0.76, 95% Cl 0.25 to 2.28, respectively).	Low
Cardio-vascular Mortality	Clozapine vs. risperidone	1 SR[1] (2 retrospective cohort studies; N =1,686)	Moderate	Unknown	Direct	Imprecise	Inconclusive: No significant differences between drugs 4.8% and 2.5%; RR 1.39, 95% CI 0.61 to 2.53	Insufficient
**Self-Harm: Suicidal Behavior, Suicide**	Clozapine versus olanzapine in high-risk patients	1 SR[1] (1 RCT; N = 980)	Low	Unknown	Direct	Imprecise	Suicidal behavior: HR 0.76, 95% CI 0.58 to 0.97;	Low
Self-Harm: Suicidal Behavior, Suicide	Clozapine vs. olanzapine in high-risk patients	1 SR[1] (1 RCT; N = 980)	Low	Unknown	Direct	Precise	Worsening on CGI-Suicide Severity: HR 0.78, 95% CI 0.61 to 0.99	Moderate
Self-Harm: Suicidal Behavior, Suicide	Clozapine vs. olanzapine in high-risk patients	1 SR[1] (1 RCT; N = 980)	Low	Unknown	Direct	Imprecise	Suicide deaths: No significant differences (5 clozapine, 3 olanzapine)	Low

Outcome	Comparators	Number of Studies Number of Subjects	Study Limitations	Consistency	: Directness	Precision	Magnitude of Effect: Summary Effect Size (95% CI)	Strength of Evidence (High, Moderate, Low, Insufficient)
Self-Harm: Suicidal Behavior, Suicide	Clozapine, risperidone, olanzapine, quetiapine, ziprasidone, aripiprazole	1 SR[1] (2 retrospective cohorts; N = 16,584)	Moderate	Consistent	Direct	Precise	Death by suicide lower with clozapine: OR 0.29 (0.14 to 0.63) compared with no treatment at 6 months and clozapine (1.1%) lower than baseline (2.2%) or other drugs (range 2.1% to 3.7%) at 1 year.	Low
Self-Harm: Suicidal Behavior, Suicide	Clozapine, risperidone, olanzapine, quetiapine, ziprasidone, aripiprazole	1 SR[1] (1 prospective cohort; N = 10,204)	High	Unknown	Direct	Precise	Suicide attempts (6 months: No statistically significant difference between drugs	Insufficient
Self-Harm: Suicidal Behavior, Suicide	Clozapine, risperidone, olanzapine, quetiapine, ziprasidone, aripiprazole	1 SR[1] (1 prospective cohort; N = 20,489)	High	Unknown	Direct	Precise	Inconclusive:<br>Suicide attempts or death by suicide: aripiprazole vs. others combined HR 0.69, 95% CI 0.42 to 1.14	Insufficient
**Core Illness Symptoms**	Oral SGAs (except carpipramine)	(212 RCTs)[13]	Moderate	Consistent	Indirect	Precise	Clozapine had significantly better improvement than the other drugs except olanzapine (SMDs on PANSS or BPRS -0.32 to -0.55. Olanzapine and risperidone superior to the other drugs, except for each other and paliperidone (SMDs -0.13 to -0.26). Paliperidone superior to lurasidone and iloperidone (SMD -0.17). All drugs superior to placebo (SMDs –0·33 to –0·88).	Low

Outcome	Comparators	Number of Studies Number of Subjects	Study Limitations	Consistency	: Directness	Precision	Magnitude of Effect: Summary Effect Size (95% CI)	Strength of Evidence (High, Moderate, Low, Insufficient)
Core Illness Symptoms	Treatment resistant patients: clozapine, risperidone, olanzapine, quetiapine, and ziprasidone	(40 RCTs)[14]	Moderate	Consistent	Indirect	Precise	The only significant difference was that the mean change in the PANSS greater with olanzapine than quetiapine (SMD -0.29, 95% CI -0.56 to -0.13).	Low
Core Illness Symptoms	Brexpiprazole vs. aripiprazole	(N=97)[15]	Moderate	Unknown	Indirect	Imprecise	Inconclusive:<br>PANSS scale -22.9 vs. -19.4 from baseline; direct comparison not reported.	Insufficient
**Overall/Any Adverse Events**								
Overall/Any Adverse Events	Asenapine vs. olanzapine	5 RCTs (4 publications); N = 2189)[7,8,16,17]	Moderate	Consistent	Direct	Precise	Pooled RR 1.00 (95% CI 0.96 – 1.05); I^2 9%	Moderate
Overall/Any Adverse Events	Quetiapine vs. risperidone	7 RCTs (N = 3254)[11,12,18-22]	Moderate	Consistent	Direct	Precise	Pooled 1.04 (95% CI 0.97 to 1.12); I^2 56%	Moderate
Overall/Any Adverse Events	Clozapine vs. olanzapine	2 RCTs (N = 182)[6,20]	Moderate	Consistent	Direct	Imprecise	Pooled RR 1.15 (95% CI = 1.00 to 1.33); I^2 0%	Low
Overall/Any Adverse Events	Risperidone vs. olanzapine	5 RCTs (N = 873)[20-24]	Moderate	Inconsistent	Direct	Precise	Pooled = RR 1.02 (95% CI = 0.81 to 1.29); I^2 = 77%	Low
Overall/Any Adverse Events	Olanzapine vs. ziprasidone	5 RCTs (N = 1097; 6 weeks to 6 months durations)[25-29]	Moderate	Inconsistent	Direct	Precise	Pooled RR 1.00 (95% CI = 0.86 to 1.16); I^2 = 80%	Low
Overall/Any Adverse Events	Olanzapine vs. quetiapine	3 RCTs (N = 448)[20-22]	Moderate	Consistent	Direct	Imprecise	Pooled RR 0.90 (95% CI 0.74 to 1.11); I^2 = 30%	Low

Outcome	Comparators	Number of Studies Number of Subjects	Study Limitations	Consistency	: Directness	Precision	Magnitude of Effect: Summary Effect Size (95% CI)	Strength of Evidence (High, Moderate, Low, Insufficient)
Overall/Any Adverse Events	Quetiapine ER versus quetiapine IR and risperidone; risperidone versus clozapine and aripiprazole; olanzapine versus paliperidone; risperidone LAI versus paliperidone and paliperidone palmitate monthly LAI; and aripiprazole versus aripiprazole monthly LAI. Additionally there were six trials comparing asenapine and olanzapine	1 SR (28 RCTs; N = 7810)	Moderate	Consistent	Direct	Imprecise	No statistically significant differences were found in each comparison.	Low

Outcome	Comparators	Number of Studies Number of Subjects	Study Limitations	Consistency	: Directness	Precision	Magnitude of Effect: Summary Effect Size (95% CI)	Strength of Evidence (High, Moderate, Low, Insufficient)
Overall/Any Adverse Events	Oral aripiprazole versus brexpiprazole, olanzapine, paliperidone, and risperidone LAI; ziprasidone versus clozapine, risperidone, iloperidone and lurasidone; risperidone versus asenapine, carpipramine and risperidone LAI; clozapine versus quetiapine, quetiapine versus risperidone LAI; olanzapine versus olanzapine LAI and lurasidone; aripiprazole monthly LAI versus paliperidone; and paliperidone palmitate monthly LAI versus 3-monthly LAI.	1 SR (31 RCTs; N = 6700)	Moderate	Unknown	Direct	Imprecise	No statistically significant differences were found in single studies of each comparison.	Insufficient

Outcome	Comparators	Number of Studies Number of Subjects	Study Limitations	Consistency	: Directness	Precision	Magnitude of Effect: Summary Effect Size (95% CI)	Strength of Evidence (High, Moderate, Low, Insufficient)
**Discontinu-ation Due to Adverse Events**	Network meta-analysis of aripiprazole, aripiprazole monthly LAI, asenapine, brexipiprazole, cariprazine, clozapine, iloperidone, lurasidone10, olanzapine, olanzapine LAI, paliperidone 3-month LAI, paliperidone, paliperidone Monthly LAI, quetiapine ER, quetiapine IR, risperidone, risperidone LAI, ziprasidone	89 RCTs (N = 29,678)	Moderate	Consistent	Indirect	Precise	LAI risperidone had statistically significantly lower risk of withdrawals due to adverse events than asenapine (OR 0.50, 95% CI 0.23,0.97); clozapine (OR 0.26, 95% CI 0.10,0.67); lurasidone (OR 0.38, 95% CI 0.17,0.79); paliperidone (OR 0.43, 95% CI 0.17,0.98); paliperidone LAI monthly (OR 0.51, 95% CI 0.26,0.98); quetiapine ER (OR 0.42, 95% CI 0.21,0.78); risperidone (OR 0.48, 95% CI 0.23,0.92); and ziprasidone (OR 0.39, 95% CI 0.18,0.76). Olanzapine had lower risk than clozapine (OR 0.40, 95% CI 0.21,0.79); lurasidone (OR 0.58, 95% CI 0.36,0.98); quetiapine IR (OR 0.64, 95% CI 0.45,0.93); risperidone (OR 0.74, 95% CI 0.55,0.98); and ziprasidone (OR 0.59, 95% CI 0.43,0.84). Aripiprazole had lower risk than ziprasidone (OR 0.65, 95% CI 0.44,0.95) and iloperidone had lower risk than clozapine (OR 0.35, 95% CI 0.13,0.91).	Low

BPRS=Brief Psychiatric Rating Scale, CATIE=clinical Antipsychotic Trials of Intervention Effectiveness, CGI-S=Clinical Global Impression - Severity, CI=confidence interval, ER=efficacy ratio, GAF=global assessment of functioning, HR=hazard ratio, LAI=long acting injectable, NR=normal range, NS=not significant, NSD=no significant difference, OR=odds ratio, PSP=, Q=Cochran's Q test, QLS=quality of life scale, RCT=randomized controlled trial, RR=relative risk, SF=short form, SFS=Social Functioning Scale, SGA=second-generation antipsychotic, SMD=standard mean difference, SOFA=Social and Occupational Functioning Assessment, SQLS=Schizophrenia Quality of Life Scale, SR=systematic review, WMD=weighted mean difference

Table 2. Strength of evidence: first-generation antipsychotic versus second-generation antipsychotic

Outcome	Comparators	Number of Studies Number of Subjects	Study Limitations	Consistency	Directness	Precision	Magnitude of Effect: Summary Effect Size (95% CI)	Strength of Evidence (High, Moderate, Low, Insufficient)
**Function: General**	Haloperidol vs. olanzapine	1 SR (1 RCT)[30] N=208	Moderate	Unknown	Direct	Imprecise	Inconclusive: GAF effect estimate: -4.00; 95% CI -13.70 to 5.70	Insufficient
**Function: Encounters with Legal System**	Haloperidol vs. olanzapine	1 SR (1 RCTs)[30] N=31	Moderate	Unknown	Direct	Imprecise	Inconclusive: Encounters with legal system: RR 3.20, 95% CI 0.76 to 13.46	Insufficient
**Function: Employment**	Haloperidol vs. risperidone	1 SR[30] (1 RCT) N=100	Moderate	Unknown	Direct	Imprecise	Inconclusive: Proportion of patients with economic independence: RR 0.94; 95% CI 0.68 to 1.29	Insufficient
Function: Employment	Perphenazine vs. olanzapine	1 SR[30] (1 RCT) N=597	Moderate	Unknown	Direct	Imprecise	Inconclusive: Proportion with paid employment: RR 1.29; 95% CI 0.70 to 2.38	Insufficient
Function: Employment	Perphenazine vs. quetiapine	1 SR[30] (1 RCT) N=598	Moderate	Unknown	Direct	Imprecise	Inconclusive: Proportion with paid employment: RR 1.75; 95% CI 0.90 to 3.43	Insufficient
Function: Employment	Perphenazine vs. risperidone	1 SR[30] (1 RCT) N=602	Moderate	Unknown	Direct	Imprecise	Inconclusive: Proportion with paid employment: RR 1.38; 95% CI 0.74 to 2.57	Insufficient
Function: Employment	Perphenazine vs. ziprasidone	1 SR[30] (1 RCT) N=446	Moderate	Unknown	Direct	Imprecise	Inconclusive: Proportion with paid employment: RR 1.22; 95% CI 0.60 to 2.51	Insufficient
**Quality of Life**	Haloperidol vs. olanzapine	1 SR[30] (5 RCTs) N=816	Moderate	Consistent	Direct	Precise	Inconclusive: Effect sizes ranged from -3.62 to 0 using different measures; CIs were not significant	Moderate

Outcome	Comparators	Number of Studies Number of Subjects	Study Limitations	Consistency	Directness	Precision	Magnitude of Effect: Summary Effect Size (95% CI)	Strength of Evidence (High, Moderate, Low, Insufficient)
Quality of Life	Haloperidol vs. quetiapine	1 SR[30] (1 RCT) N=207	Moderate	Unknown	Direct	Imprecise	Inconclusive: Effect estimate 0.00; 95% CI -1.38 to 1.38	Insufficient
Quality of Life	Haloperidol vs. risperidone	1 SR[30] (2 RCTs) N=352	Moderate	Inconsistent	Direct	Imprecise	Inconclusive: Effect estimates ranged from -0.10 to 0.10; CIs were not significant	Insufficient
Quality of Life	Haloperidol vs. ziprasidone	1 SR[30] (2 RCTs) N=784	High	Inconsistent	Direct	Imprecise	Studies favored ziprasidone in quality of life measures. One trial found effect favoring ziprasidone based on QLS (effect estimate 12.12; 95% CI -22.06 to -2.17); there was no difference in another trial in MANSA (effect estimate -0.10; 95% CI -1.48 to 1.28)	Low
Quality of Life	Perphenazine vs. aripiprazole	1 SR[30] (1 RCT) N=300	Moderate	Unknown	Direct	Imprecise	Inconclusive: Proportion with 20% improvement: RR 4.74; 95% CI 2.58 to 8.69	Insufficient
Quality of Life	Perphenazine vs. olanzapine	1 SR[30] (1 RCT) N=597	Moderate	Unknown	Direct	Precise	No difference: Effect estimate 0.00; 95% CI -0.16 to 0.16	Low
Quality of Life	Perphenazine vs. quetiapine	1 SR[30] (1 RCT) N=598	Moderate	Unknown	Direct	Precise	No difference: Effect estimate 0.10; 95% CI -0.07 to 0.27	Low
Quality of Life	Perphenazine vs. risperidone	1 SR[30] (1 RCT) N=602	Moderate	Unknown	Direct	Precise	No difference: Effect estimate -0.07; 95% CI -0.24 to 0.10	Low

Outcome	Comparators	Number of Studies Number of Subjects	Study Limitations	Consistency	Directness	Precision	Magnitude of Effect: Summary Effect Size (95% CI)	Strength of Evidence (High, Moderate, Low, Insufficient)
Quality of Life	Perphenazine vs. ziprasidone	1 SR[30] (1 RCT) N=446	Moderate	Unknown	Direct	Precise	No difference: Effect estimate -0.07; 95% CI -0.27 to 0.13	Low
**Response**	Fluphenazine vs. olanzapine	1 SR[30] (1 RCT) N=60	Moderate	Unknown	Direct	Imprecise	Inconclusive: RR 0.74 (95% CI 0.51 to 1.07)	Insufficient
Response	Fluphenazine vs. quetiapine	1 SR[30] (1 RCT) N=25	Moderate	Unknown	Direct	Imprecise	Inconclusive: RR 0.62 (95% CI 0.12 to 3.07)	Insufficient
Response	Fluphenazine vs. risperidone	1 SR[30] (1 RCT) N=26	Moderate	Unknown	Direct	Imprecise	Inconclusive: RR 0.67 (95% CI 0.13 to 3.35)	Insufficient
Response	Haloperidol vs. aripiprazole	1 SR[30] (5 RCTs) N=2,185	Moderate	Inconsistent	Direct	Precise	No difference: RR 1.01 (95% CI 0.76 to 1.34; I^2=83%)	Low
Response	Haloperidol vs. asenapine	1 SR[30] (1 RCT) N=335	Moderate	Unknown	Direct	Imprecise	Inconclusive: RR 0.82 (95% CI 0.64 to 1.04)	Insufficient
Response	Haloperidol vs. clozapine	1 SR[30] (2 RCTs) N=144	Moderate	Inconsistent	Direct	Imprecise	Inconclusive: RR 0.64 (95% CI 0.28 to 1.47; I^2= 72%)	Insufficient
Response	Haloperidol vs. olanzapine	1 SR[30] (14 RCTs) N=4,099	Moderate	Inconsistent	Direct	Precise	RR 0.86 (95% CI 0.78 to 0.96; I^2=55%); favors olanzapine	Low
Response	Haloperidol vs. quetiapine	1 SR[30] (6 RCTs) N=1,421	Moderate	Inconsistent	Direct	Precise	No difference: RR 0.99 (95% CI 0.76 to 1.30; I^2=77%)	Low
Response	Haloperidol vs. risperidone	1 SR[30] (16 RCTs) N=3,452)	Moderate	Consistent	Direct	Precise	No difference: RR 0.94 (95% CI 0.87 to 1.02; I^2=29%)	Moderate
Response	Haloperidol vs. ziprasidone	1 SR[30] (6 RCTs) N=1,283	Moderate	Inconsistent	Direct	Imprecise	Inconclusive: RR 0.98 (95% CI 0.74 to 1.30; I^2=80%)	Low
Response	Perphenazine vs. aripiprazole	1 SR[30] (1 RCT) N=300	Moderate	Unknown	Direct	Imprecise	Inconclusive: RR 0.95 (95% CI 0.64 to 1.40)	Insufficient

Outcome	Comparators	Number of Studies Number of Subjects	Study Limitations	Consistency	Directness	Precision	Magnitude of Effect: Summary Effect Size (95% CI)	Strength of Evidence (High, Moderate, Low, Insufficient)
**Remission**	Haloperidol vs. clozapine	1 SR[30] (1 RCT) N=71	Moderate	Unknown	Direct	Imprecise	Inconclusive: RR 0.16 (95% CI 0.02 to 1.20)	Insufficient
Remission	Haloperidol vs. olanzapine	1 SR[30] (3 RCTs) N=582	Moderate	Consistent	Direct	Imprecise	RR 0.65 (95% CI 0.45 to 0.94; I^2=54%); favors olanzapine	Low
Remission	Haloperidol vs. quetiapine	1 SR[30] (1 RCT) N=207	High	Unknown	Direct	Imprecise	Inconclusive: RR 0.72 (95% CI 0.41 to 1.25)	Insufficient
Remission	Haloperidol vs. risperidone	1 SR[30] (2 RCTs) N=179	Moderate	Consistent	Direct	Imprecise	Inconclusive: RR 0.84 (95% CI 0.56 to 1.24; I^2=0%)	Low
Remission	Haloperidol vs. ziprasidone	1 SR[30] (3 RCTs) N=1,085	High	Consistent	Direct	Precise	No difference: RR 0.89 (95% CI 0.71 to 1.12; I^2= 12%)	Low
**Reduction in Self Harm**	Haloperidol vs. olanzapine	1 SR[30] (1 RCT) N=182	Moderate	Unknown	Indirect	Imprecise	Inconclusive: Attempted suicide: RR 3.13; 95% CI 0.13 to 76 and 0.64 Completed suicide: RR 3.13; 95% CI 0.13 to 76 and 3.86	Insufficient
Reduction in Self Harm	Perphenazine and olanzapine	1 SR[30] (1 RCT) N=597	Moderate	Unknown	Indirect	Imprecise	Inconclusive: Attempted suicide: RR 0.64; 95% CI 0.06 to 7.06 Completed suicide: RR 3.86; 95% CI 0.40 to 37	Insufficient
**Overall Adverse Events**	Haloperidol vs. aripiprazole	1 SR[30] (3 RCTS) N=1,713	Moderate	Consistent	Direct	Precise	RR 1.11; 95% CI 1.06 to 1.17; I^2=0%; less with aripiprazole	Moderate
Overall Adverse Events	Haloperidol vs. risperidone	1 SR[30] (8 RCTs) N=1,313	Moderate	Consistent	Direct	Precise	RR 1.20; 95% CI 1.01 to 1.42; I^2= 84%; less with risperidone	Moderate
Overall Adverse Events	Haloperidol vs. ziprasidone	1 SR[30] (6 RCTs) N=1,448	Moderate	Consistent	Direct	Precise	RR 1.13; 95% CI 1.03 to 1.23; I^2=31%; less with ziprasidone	Moderate

Outcome	Comparators	Number of Studies Number of Subjects	Study Limitations	Consistency	Directness	Precision	Magnitude of Effect: Summary Effect Size (95% CI)	Strength of Evidence (High, Moderate, Low, Insufficient)
**Withdrawal Due to Adverse Events**	Fluphenazine vs. olanzapine	1 SR[30] (1 RCT)<br>N=60	Moderate	Unknown	Indirect	Imprecise	Inconclusive:<br>RR 0.74; 95% CI 0.51 to 1.07	Insufficient
	Fluphenazine vs. quetiapine	1 SR[30] (1 RCT)<br>N=25	Moderate	Unknown	Indirect	Imprecise	Inconclusive:<br>RR 0.19; 95% CI 0.01 to 3.52	Insufficient
Withdrawal Due to Adverse Events	Haloperidol vs. asenapine	1 SR[30] (1 RCT)<br>N=335	Moderate	Unknown	Indirect	Imprecise	Inconclusive:<br>RR 1.53; 95% 0.74 to 3.16	Insufficient
Withdrawal Due to Adverse Events	Haloperidol vs. aripiprazole	1 SR[30] (7 RCTs) + 1 additional RCT<br>N= 3,232	Moderate	Consistent	Direct	Precise	RR 1.25; 1.07 to 1.47; I^2=0%	Moderate
Withdrawal Due to Adverse Events	Haloperidol vs. clozapine	1 SR[30] (5 RCTs)<br>N=719	Moderate	Consistent	Direct	Imprecise	Inconclusive:<br>RR 1.00; 95% CI 0.66 to 1.50; I^2=0%	Low
Withdrawal Due to Adverse Events	Haloperidol vs. olanzapine	1 SR[30] (21 RCTs) + 3 RCTs<br>N= 5,708	Moderate	Consistent	Direct	Precise	RR 1.89; 95% CI 1.57 to 2.27; I^2=0%	Moderate
Withdrawal Due to Adverse Events	Haloperidol vs. quetiapine	1 SR[30] (8 RCTs) + 2 RCTs<br>N=1,759	Moderate	Consistent	Direct	Imprecise	Inconclusive:<br>RR 1.97; 95% CI 0.96 to 4.01; I^2=62%	Low
Withdrawal Due to Adverse Events	Haloperidol vs. risperidone	1 SR[30] (23 RCTs) + 2 RCTs<br>N=4,581	Moderate	Consistent	Direct	Precise	RR 1.32; 95% CI 1.09 to 1.60; I^2=0%	Moderate
Withdrawal Due to Adverse Events	Haloperidol vs. ziprasidone	1 SR[30] (6 RCTs) + 1 RCT<br>N=1597	Moderate	Consistent	Direct	Precise	RR 1.68; 95% CI 1.26 to 2.23; I^2=0%	Moderate

Outcome	Comparators	Number of Studies Number of Subjects	Study Limitations	Consistency	Directness	Precision	Magnitude of Effect: Summary Effect Size (95% CI)	Strength of Evidence (High, Moderate, Low, Insufficient)
Withdrawal Due to Adverse Events	Perphenazine vs. aripiprazole	1 SR[30] (1 RCT) N=300	Moderate	Unknown	Direct	Imprecise	Inconclusive: RR 0.53; 95% CI 0.27 to 1.05	Insufficient
Withdrawal Due to Adverse Events	Perphenazine vs. olanzapine	1 SR[30] (1 RCT) N=597	Moderate	Unknown	Direct	Imprecise	Inconclusive: RR 0.83; 95% CI 0.58 to 1.19	Insufficient
Withdrawal Due to Adverse Events	Perphenazine vs. quetiapine	1 SR[30] (1 RCT) N=598	Moderate	Unknown	Direct	Imprecise	Inconclusive: RR 1.05; 95% CI 0.72 to 1.55	Insufficient
Withdrawal Due to Adverse Events	Perphenazine vs. risperidone	1 SR[30] (1 RCT) N=602	Moderate	Unknown	Direct	Imprecise	Inconclusive: RR 1.54; 95% CI 1.00 to 2.36	Insufficient
Withdrawal Due to Adverse Events	Perphenazine vs. ziprasidone	1 SR[30] (1 RCT) N=446	Moderate	Unknown	Direct	Imprecise	Inconclusive: RR 1.01; 95% CI 0.65 to1.58	Insufficient

CI=confidence interval, OR=odds ratio, RCT=randomized controlled trial, SR=systematic review
